# Modelling cardiac fibrosis using three-dimensional cardiac microtissues derived from human embryonic stem cells

**DOI:** 10.1186/s13036-019-0139-6

**Published:** 2019-02-13

**Authors:** Mi-Ok Lee, Kwang Bo Jung, Seong-Jae Jo, Sung-Ae Hyun, Kyoung-Sik Moon, Joung-Wook Seo, Sang-Heon Kim, Mi-Young Son

**Affiliations:** 1 Stem Cell Convergence Research Center, Korea Research Institute of Bioscience and Biotechnology (KRIBB), Daejeon, 341411 Republic of Korea; 2 Department of Functional Genomics, KRIBB School of Bioscience, Korea University of Science and Technology (UST), Daejeon, 34113 Republic of Korea; 3 Research Group for Safety Pharmacology, Korea Institute of Toxicology, KRICT, Daejeon, 34114 Republic of Korea; 4 Center for Biomaterials, Korea Institute of Science and Technology (KIST), Seoul, 02792 Republic of Korea; 5 Department of Biomedical Engineering, KIST school, UST, Daejeon, 34113 Republic of Korea

**Keywords:** Cardiac fibrosis, Cardiac sphere, Cardiac microtissue, Cardiomyocyte, Mesenchymal stem cell

## Abstract

**Background:**

Cardiac fibrosis is the most common pathway of many cardiac diseases. To date, there has been no suitable in vitro cardiac fibrosis model that could sufficiently mimic the complex environment of the human heart. Here, a three-dimensional (3D) cardiac sphere platform of contractile cardiac microtissue, composed of human embryonic stem cell (hESC)-derived cardiomyocytes (CMs) and mesenchymal stem cells (MSCs), is presented to better recapitulate the human heart.

**Results:**

We hypothesized that MSCs would develop an in vitro fibrotic reaction in response to treatment with transforming growth factor-β1 (TGF-β1), a primary inducer of cardiac fibrosis. The addition of MSCs improved sarcomeric organization, electrophysiological properties, and the expression of cardiac-specific genes, suggesting their physiological relevance in the generation of human cardiac microtissue model in vitro. MSCs could also generate fibroblasts within 3D cardiac microtissues and, subsequently, these fibroblasts were transdifferentiated into myofibroblasts by the exogenous addition of TGF-β1. Cardiac microtissues displayed fibrotic features such as the deposition of collagen, the presence of numerous apoptotic CMs and the dissolution of mitochondrial networks. Furthermore, treatment with pro-fibrotic substances demonstrated that this model could reproduce key molecular and cellular fibrotic events.

**Conclusions:**

This highlights the potential of our 3D cardiac microtissues as a valuable tool for manifesting and evaluating the pro-fibrotic effects of various agents, thereby representing an important step forward towards an in vitro system for the prediction of drug-induced cardiac fibrosis and the study of the pathological changes in human cardiac fibrosis.

**Electronic supplementary material:**

The online version of this article (10.1186/s13036-019-0139-6) contains supplementary material, which is available to authorized users.

## Background

Cardiac fibrosis is a common feature of most myocardial pathologies, including ischaemic cardiomyopathy, inherited cardiomyopathy mutations, metabolic syndrome, diabetes, and ageing [1, 2]. Increased mechanical stress or myocardial injury can trigger cardiac fibrosis, which might contribute to contractile and diastolic dysfunctions and subsequent sudden death [3]. Cardiac fibrosis is characterized by the excess accumulation of extracellular matrix (ECM) components, such as collagen and fibronectin, with the consequent pathological remodelling of ECM, followed by transforming fibroblasts into myofibroblasts [4, 5]. These myofibroblasts have high fibrotic activity marked by the expression of α-smooth muscle actin (α-SMA), resulting in myocardial fibrosis and stiffening, which eventually impairs cardiac function [6, 7].

The exploration of the pathogenesis and therapy development for cardiac fibrosis is hampered by the lack of appropriate experimental models that fully recapitulate human cardiac fibrosis. An improvement of in vitro cell or tissue models may open up new possibilities for disease modelling, drug discovery, and regenerative medicine by providing fast and controllable platforms with high availability and relatively low cost compared to animal models. As yet, few in vitro models of cardiac fibrosis have been developed [8–12]. Most are composed of cardiac cells derived from neonatal mouse and rat hearts and thus have less relevance to human pathophysiology. In addition, most current models of cardiac tissue contain only cardiomyocytes (CMs) and lack other key cell types found in the human heart [13]. Therefore, there is a need for an in vitro human cardiac fibrosis model that possesses the physiologically relevant cell combination and can mimic the three-dimensional (3D) nature of native cardiac tissue.

Due to the limited amount of human primary CMs available, CMs derived from human pluripotent stem cells (hPSCs), including human embryonic stem cells (hESCs) and human induced pluripotent stem cells (hiPSCs), have emerged as the most appropriate cell source for modelling the human heart in vitro, with obvious advantages of multiple functionalities and increased throughput [14, 15]. Though hPSC-derived CMs or cardiac progenitor cells represent immature phenotypes in their morphological and other physiological and biochemical properties [16], there is a clear incentive to use hPSC-derived CMs as a foundation to generate 3D human cardiac tissue that can eventually be tailored to patient-specific models of normal and diseased cardiac tissues. However, to date, there are no suitable in vitro cardiac fibrosis model based on hPSC-derived 3D cardiac tissue to study the pathological changes in human cardiac fibrosis and to evaluate novel treatments.

In this study, we developed an in vitro model of cardiac fibrosis through hESC-derived 3D cardiac microtissues, composed of CMs and mesenchymal stem cells (MSCs) differentiated from the same hESC line. In the normal adult heart, CMs represent only 30% of the total cell number and the remaining 70% consists of various types of cells, among which cardiac mesenchymal cells are in the majority [17]. Cardiac mesenchymal cells can provide a major precursor population to generate fibroblasts, which mediate scar formation via fibroblast–myofibroblast transition during fibrosis [18–23]. Therefore, to engineer a physiologically relevant in vitro cardiac microtissues model, we used MSCs to provide fibroblasts, which can be differentiated towards myofibroblasts after stimulation with pro-fibrotic agents. The molecular and cellular properties of these 3D cardiac microtissues were characterized and the pro-fibrotic consequences were observed after induction with transforming growth factor-β1 (TGF-β1) or other pro-fibrotic mediators. We believe that our disease model using 3D cardiac microtissues may be a suitable in vitro model for studying fibrotic changes in human heart tissue and can potentially contribute to the development of more physiologically relevant preclinical drug discovery platforms.

## Results

### Differentiation and characterization of CMs from hESCs

**Fig. 1:**
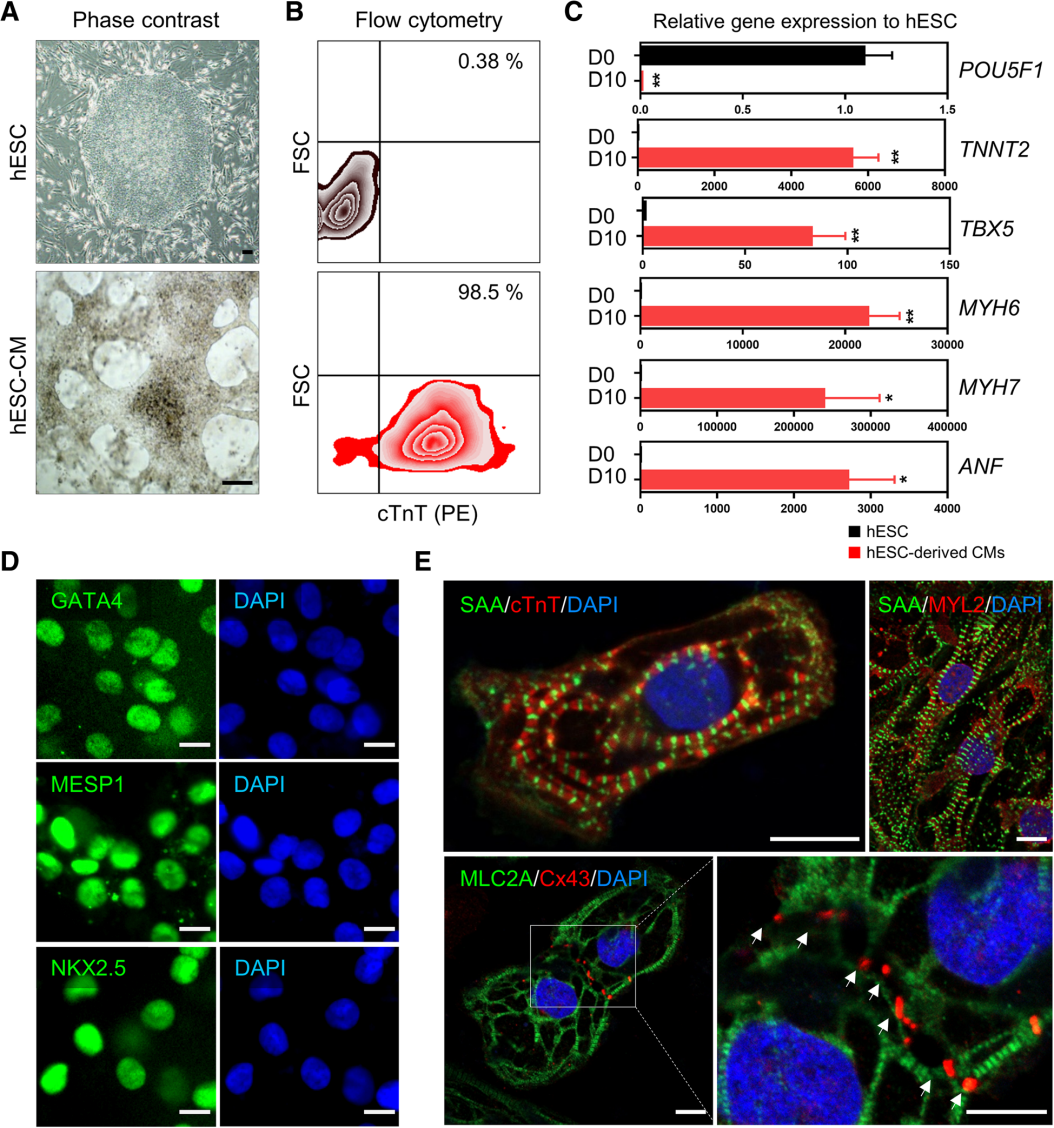
Characterization of CMs differentiated from hESCs. **a** Representative morphology of undifferentiated hESCs and CMs differentiated from hESCs after 10 days. Scale bars, 100 μm. **b** Flow cytometry plots of cardiac-specific marker cardiac troponin T (cTnT)^+^ cells. **c** Quantitative RT-PCR of hESC-specific marker (POU5F1) and CM-specific markers (TNNT2, TBX5, MYH6, MYH7, and ANF) in undifferentiated hESCs and differentiated CMs*.* Data are the means±SD of three independent experimental replicates (*n* = 3). **p < 0.01 **d** Representative immunofluorescent staining of hESC-derived CMs for cardiac-specific transcription factors (GATA4, MESP1, and NKX2.5). Scale bars, 10 μm. **e** Immunofluorescent staining for sarcomeric proteins (sarcomeric-alpha actinin (SAA), cTnT, MYL2, and MLC2A) and junctional protein (Cx43). Nuclei were stained with DAPI (blue). White arrow indicated Cx43-positive gap junction. Scale bars, 10 μm

To obtain human CMs for the development of a human cardiac microtissue model, we differentiated hESCs, which can provide an unlimited source of starting cells for CM differentiation, into CMs by manipulating Wnt signalling as previously described [24]. At 8 days after the induction of differentiation, the synchronous and spontaneous beating areas were observed (Fig. 1a and Additional file 1: Movie 1). FACS analysis with CM-specific markers (cardiac muscle Troponin T (cTnT)) showed that hESCs were differentiated into CMs with high purity (> 98%) (Fig. 1b). The relative expression of *POU5F1*, a gene encoding an hESC-specific transcription factor, in differentiated CMs was markedly reduced compared with undifferentiated hESCs (Fig. 1c). By contrast, the expression of genes involved in the formation of sarcomere, the basic unit of myofibrils, including *TNNT2*, *MYH6*, and *MYH7*, and the peptide hormone gene (*ANF*) secreted in the cardiac muscle were significantly increased (Fig. 1c). We confirmed the expression of cardiac transcription factors (GATA4, MESP1, NKX2.5) in the nucleus (Fig. 1d), and further examined the formation of a myofibril structure responsible for contraction and relaxation of CMs and cardiac sarcomere by immunofluorescence for cTnT, MYL2, MLC2A, and sarcomeric alpha-actinin (SAA) (Fig. 1e). We showed the expression of the gap junctional protein Connexin 43 (Cx43), which plays an important role in the electrical coupling of myocardium [25], in the region of cell–cell interactions (Fig. 1e). Myofibrils stained with MLC2A were connected between the two cells via Cx43 (Fig. 1e). These results showed that CMs differentiated from hESCs were interconnected via Cx43-mediated gap junctions, which have a similar structure to the Cx43 gap junction plaque in the intercalated disc of cardiac muscle [26]. Overall, these data demonstrated that hESC differentiated into CMs with high purity and expressed cardiac function-related proteins, implying that hESC-derived CMs are appropriate cell sources for generation of an in vitro human cardiac tissue model.

### Generation of 3D human cardiac microtissues

In this study, we attempted to simulate the pathological characteristics of cardiac fibrosis by introducing the 3D culture method. When 50,000 hESC-derived single CMs were seeded on round bottom 96-well ultra-low attachment plates, self-aggregation occurred slowly, resulting in uniform-sized cell spheroids (approximately 500 μm in diameter) (Additional file 2: Figure S1A). The created cardiac spheroids were beating at regular intervals and were maintained for more than 2 months (Additional file 3: Movie 2). TGF-β1 signaling has been shown to play important roles in mediating fibrotic responses by regulating ECM remodelling and excessive collagen deposition, which eventually results in cardiac hypertrophy and fibrosis [27]. Therefore, we tested whether cardiac fibrotic phenotypes can be reproduced by treating cardiac spheroids with TGF-β1. However, TGF-β1 treatment for 3 weeks did not show any marked effect on collagen deposition in the cardiac spheroids (Additional file 2: Figure S1B).

**Fig. 2:**
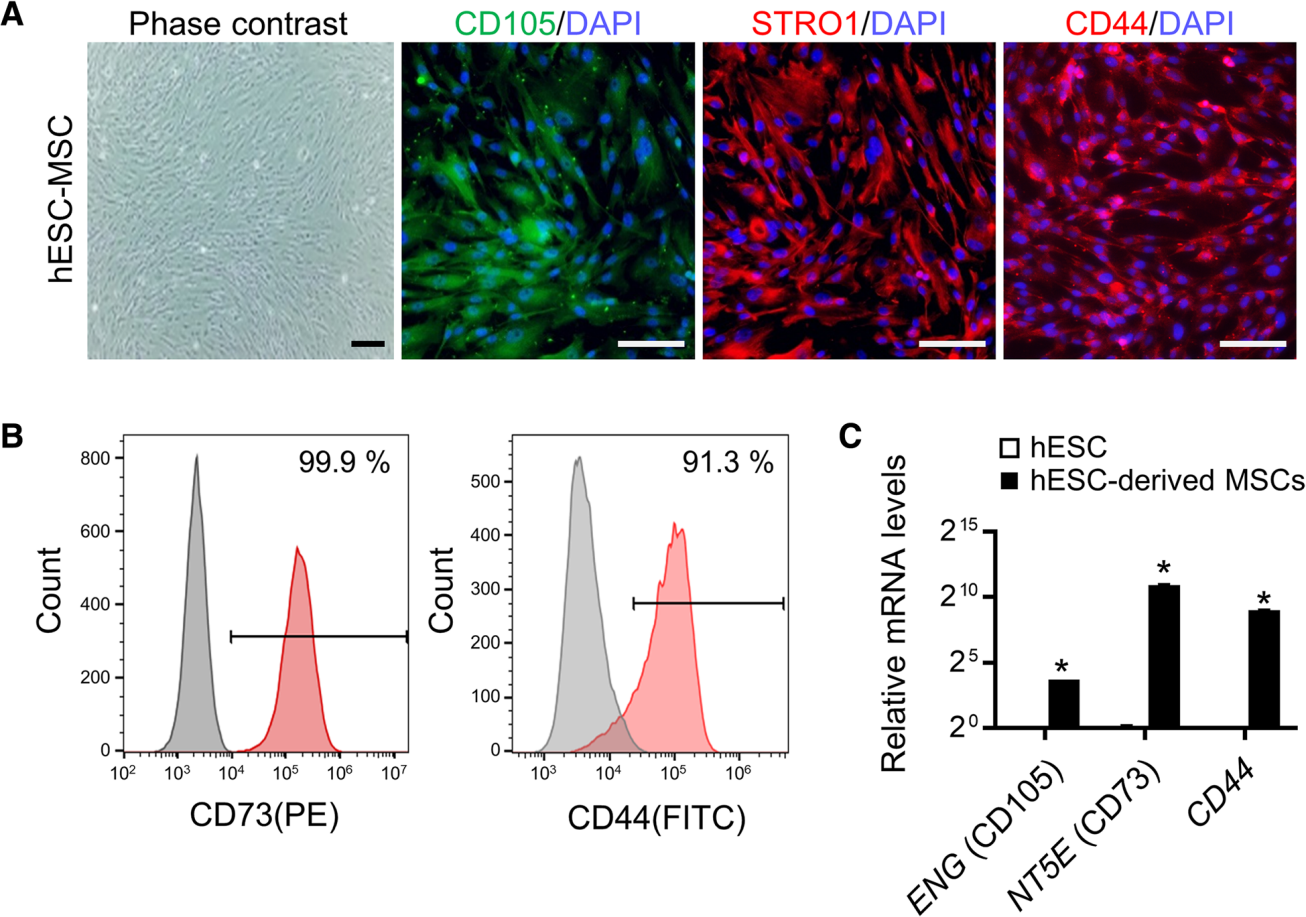
Characterization of MSCs derived from hESCs. **a** Representative morphology of differentiated MSCs and immunofluorescence staining for MSC-specific markers (CD105, STRO1, and CD44). Nuclei were stained with DAPI (blue). Scale bars, 100 μm. **b** Histograms of flow cytometry analysis for MSC surface markers (CD73 and CD44). The percentage of CD73^+^ and CD44^+^ cells in the total cell population. (**c**) qRT-PCR analysis of MSC markers (Endoglin (ENG; CD105), Ecto-5-prime-nucleotidase (NT5E; CD73), and CD44) in undifferentiated hESCs and MSCs differentiated from hESCs. Data are the means±SD of three independent experimental replicates (*n* = 3). **p < 0.01

To establish a microtissue model that ultimately aimed to closely recapitulate human cardiac fibrosis, we used MSCs as a source of fibroblasts, which can be obtained by differentiation from the same hESCs used in differentiating into CMs. The expression of MSC surface markers such as CD105 and STRO-1 was confirmed by immunostaining and over 99% of the differentiated MSCs were in the CD73-positive populations (Fig. 2a, b). Most importantly, we found that most hESC-derived MSCs expressed CD44, a hyaluronic acid receptor and marker of cardiac MSCs [21], as evaluated by immunofluorescence and FACS analysis (Fig. 2a, b). qRT-PCR also confirmed that these differentiated MSCs expressed relevant markers, such as *ENG* (CD105), *NT5E* (CD73), and *CD44* (Fig. 2c). It has been previously reported that endogenous CD44-positive MSCs contribute to the fibroblast population in myocardial infarction [18].

**Fig. 3:**
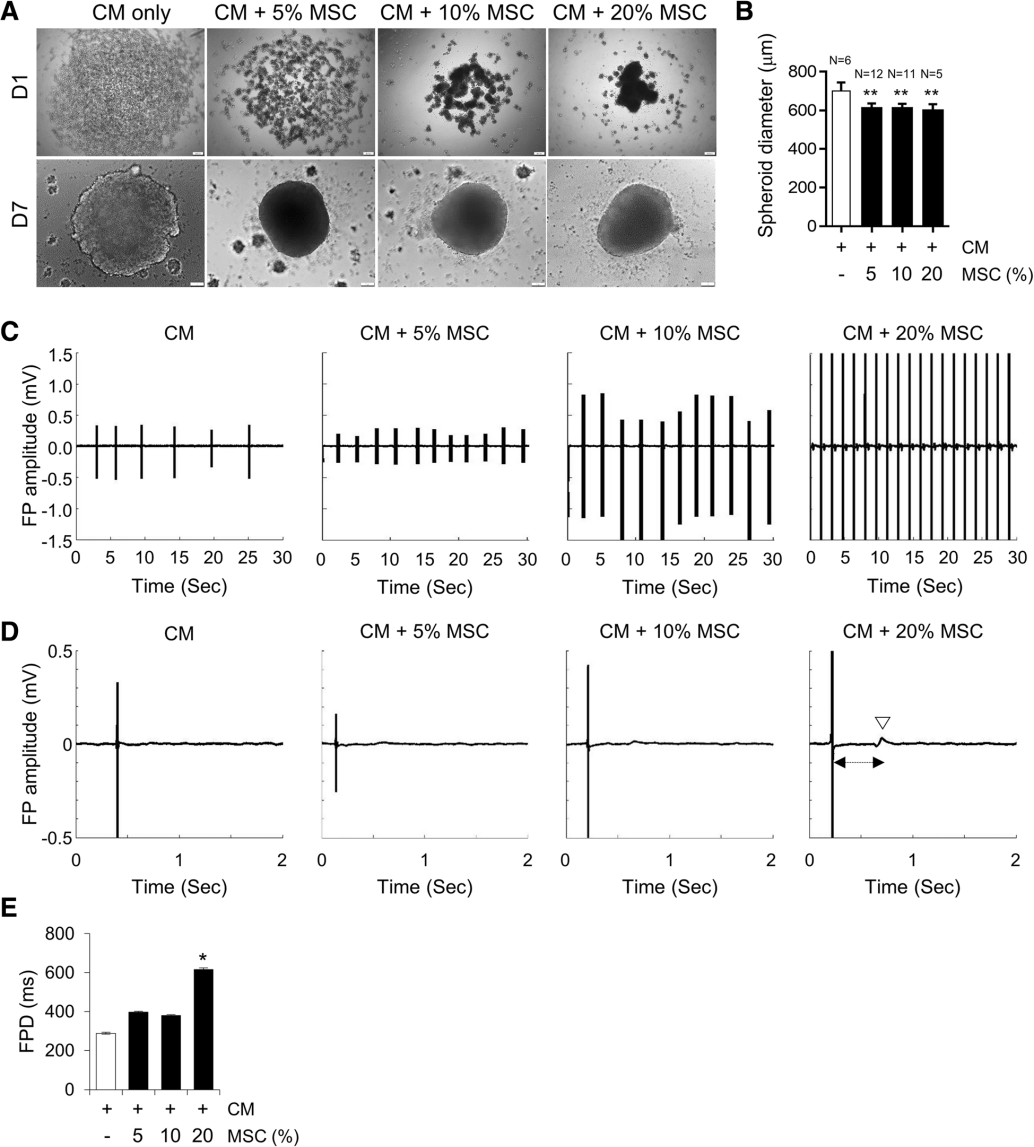
Importance of MSCs in generating 3D cardiac microtissue. (**a**) Representative image of CMs and MSCs differentiated from the same hESC line when co-cultured as 3D spheroids for day 1 and 7. Cardiac spheroid formation was dependent on the ratio of MSCs to CMs. Scale bars, 100 μm. (**b**) Average diameters of cardiac spheroids. Data are the means±SD of replicates (*n* ≥ 5). **p < 0.01. (**c**) Representative raw traces of the microelectrode array (MEA) recording from control CMs and CMs co-cultured with 5, 10 and 20% MSCs on day 6 after seeding. (**d**) The representative traces of averaged field potential (FP) recorded by MEA in the control CMs (*n* = 14) and CMs co-cultured with 5% (*n* = 37), 10% (*n* = 49) and 20% MSCs (*n* = 48). Open triangle indicates the peak of FP and bilateral arrow indicates the field potential duration (FPD). (**e**) FPD obtained from the control CMs (*n* = 14) and CMs co-cultured with 5% (*n* = 37), 10% (*n* = 49) and 20% MSCs (*n* = 48). **p < 0.01, *p < 0.05

Therefore to develop a reliable 3D cardiac tissue model with increased physiological relevance, hESC-derived CMs were mixed with hESC-derived MSCs similar to those likely to be found in human cardiac tissue. In consideration of a report that cardiac fibroblasts account for 20% of total mass of the myocardium [28, 29] and the considerable variation of the ratio of cell numbers of fibroblasts in heart across studies [30], we tested various ratios of MSCs (5–20%), which are used as a source of fibroblasts in CM-MSC cardiac microtissues. Therefore, a single cell suspension of CMs was mixed at a 5–20% ratio of a single cell suspension of MSCs in round bottom 96-well ultra-low attachment plates to form cardiac microtissues called spheroids. Mixing with MSCs, known ECM-secreting cells, stimulated concentration-dependent formation of self-aggregates into cardiac microtissues (Fig. 3a). The average size of cardiac spheroids comprising hESC-derived CMs and 20% MSCs (597.32 μm ± 7.55) was significantly smaller than that of CM only spheroids (700.00 μm ± 22.65) (Fig. 3b). Cardiac spheroids comprising hESC-derived CMs and 20% MSCs also showed regular contractile activity (Additional file 4: Movie 3). To elucidate the electrophysiological features, we used an MEA system that enables simultaneous, accurate, and real-time recordings from multiple sites in the cell network [31]. MEA analyses revealed that CMs co-cultured with 20% MSCs showed a more regular spontaneous beating pattern with a beating frequency of approximately 49.15 ± 5.66 beats/min at 0 to 1 min than control CMs (17.81 ± 2.80 beats/min), CMs co-cultured with 5% (17.3 ± 0.63 beats/min) or 10% MSCs (26.1 ± 4.24 beats/min) (Fig. 3c). Furthermore, we observed a higher amplitude of field potential (FP) from CMs co-cultured with 20% MSCs compared with control CMs and CMs co-cultured with 5% or 10% MSCs (Fig. 3d). The field potential duration (FPD) of CMs co-cultured with 20% MSCs (616.82 ± 55.91 ms) was significantly increased compared to controls (288.66 ± 19.42 ms), CMs co-cultured with 5% (397.77 ± 20.81 ms) or 10% MSCs (380.23 ± 28.78 ms) (Fig. 3e), suggesting that the addition of 20% MSCs into 3D cardiac microtissues may promote functional improvement.

### Characterization of 3D human cardiac microtissues

**Fig. 4:**
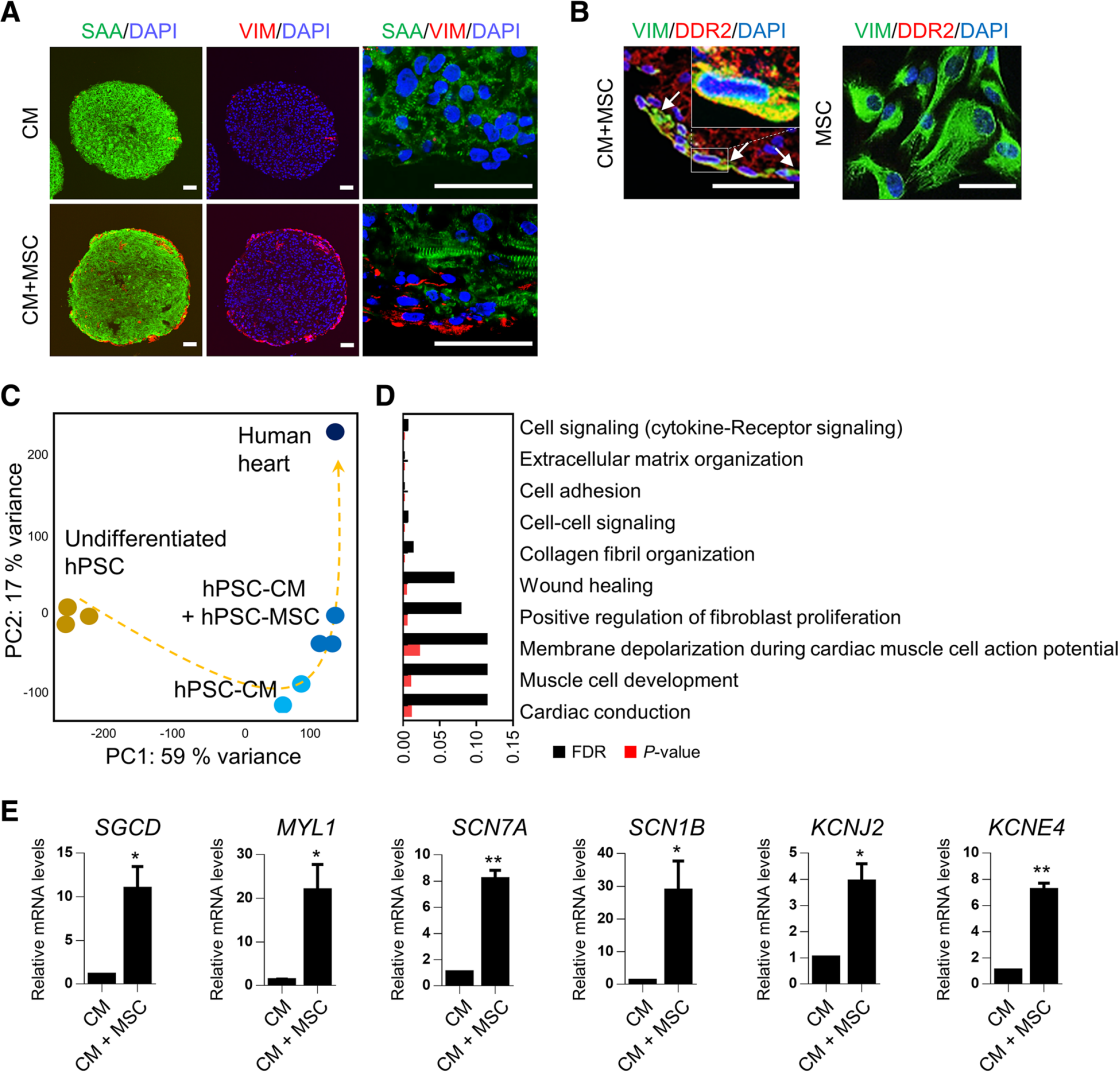
Cellular and molecular assessment of 3D cardiac microtissue. **a** Immunofluorescent staining for cardiac-specific marker (sarcomeric-alpha actinin; SAA) and MSC/pan-fibroblast-specific marker (vimentin; VIM) to visualize cell distribution in CM-MSC cardiac microtissue on day 14. Nuclei were stained with DAPI (blue). Scale bars, 50 μm. **b** Cardiac microtissue sections were co-immunostained with anti-vimentin (green) for MSC/pan-fibroblast-specific marker and anti-DDR2 (red) for the cardiac fibroblast marker (left panel). Cells positive for both vimentin/DDR2 occurred within cardiac microtissue (arrows), suggesting a transdifferentiation of MSCs into fibroblasts. hESC-derived MSCs were analyzed by immunofluorescence using anti-vimentin (green) and anti-DDR2 (red) (right panel). **c** Principal component analysis (PCA) based on total gene expression in undifferentiated hESC (*n* = 3), hESC derived CMs (hESC-CM) (*n* = 2), CM-MSC cardiac microtissue (hESC-CM + hESC-MSC) (*n* = 3), and human heart (*n* = 1). **d** Reactome pathway terms enriched in up-regulated DEGs (> 2-fold change) in the transcriptome of CM-MSC cardiac microtissues, compared to that of hESC-CM. **e** qRT-PCR analysis of cardiac function-related genes, including *SGCD*, *MYL1*, *SCN7A*, *SCN1B*, *KCNJ2*, and *KCNE4.* Data are the means±SD of three independent experimental replicates (*n* = 3).**p < 0.01

A comprehensive assessment of morphological integrity and cellular composition was conducted over cardiac microtissues by using immunofluorescence of key cell type-specific markers and cardiac gene expression analysis. Cardiac spheroids comprising CMs in combination with MSCs (CM-MSC cardiac microtissues) showed a compartmentalized organization in which SAA-positive CMs were concentrated in the center of the spheroid (Fig. 4a), while MSCs that were positive for vimentin, a mesenchymal and pan-fibroblastic marker, were at the periphery of the spheroid (Fig. 4a). Cells in the periphery of the spheroid also expressed the cardiac fibroblast-specific marker DDR2 (Fig. 4b, left panel), a collagen receptor expressed early in the adult heart and in development [32], which was not expressed in hESC-derived MSCs (Fig. 4b, right panel). Furthermore, vimentin and DDR2 double-positive cells emerged, suggesting a transdifferentiation of MSCs into fibroblasts within CM-MSC cardiac microtissues (Fig. 4b, arrows in left panel). Using PCA, we found that global transcriptomic data of CM-MSC cardiac microtissues clustered independently of control cardiac microtissues comprising only CMs, which were isolated from undifferentiated hESCs on principal component 1 (PC1, 51% variance), and became more similar to the transcriptome of the human heart (Fig. 4c). In addition, the transcriptome of the CM-MSC cardiac microtissues was distinct from that of CMs and tended to be closer to the transcriptome of human heart on principal component 2 (PC2, variance 26%) (Fig. 4c). Further analysis of the transcriptomes revealed 746 differentially expressed genes (DEGs) in CM-MSC cardiac microtissues compared with control cardiac microtissues without MSCs. As expected, in the gene-set enrichment with increased genes in CM-MSC cardiac microtissues, a set of genes related to fibroblast function, such as cell signalling, com, and wound healing was predominantly identified (Fig. 4d). Consistently, pathways associated with cardiac muscle function, such as cardiac conduction and membrane depolarization during cardiac muscle cell action potential were also enriched in the DEG analysis (Fig. 4d). To confirm the regulation of genes related to cardiac muscle function by mesenchyme, the expression of the related genes was confirmed by qRT-PCR. In agreement with the microarray results, there was increased expression of genes related to cardiac function such as *SGCD*, which plays a role in dystrophin complex stabilization, *MYL1*, encoding the myosin light chain involved in cardiac conduction, and *SCN7A*, *SCN1B*, *KSNJ2*, and *KCNE4*, encoding voltage-gated ion channel related proteins (Fig. 4e). These results suggest that CM-MSC cardiac microtissues not only acquired fibroblastic functions by changing cellular composition but might also improve cardiac function through myocyte-mesenchyme interactions.

### TGF-β1-induced fibrosis in CM-MSC cardiac microtissues

**Fig. 5:**
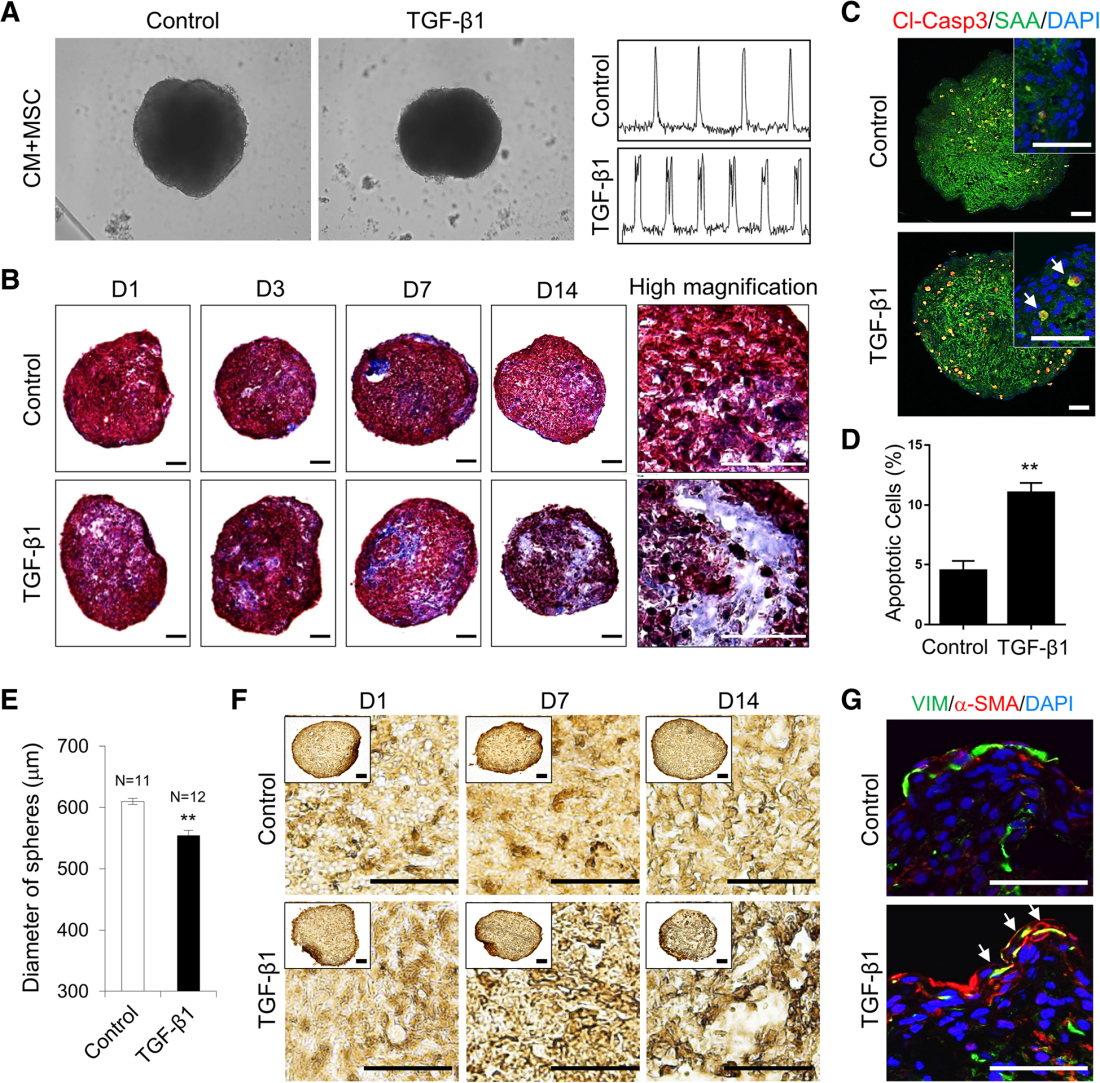
Recapitulation of phenotypes observed in TGF-β1-induced cardiac fibrosis using 3D cardiac microtissue. **a** Representative images of CM-MSC cardiac microtissue with or without 5 ng/ml TGF-β1 treatment for 2 weeks (left panel). Representative trace obtained by plotting z-axis profile of cardiac microtissue beating (right panel). **b** Masson’s Trichrome staining of CM-MSC cardiac microtissue sections at serial time points after TGF-β1 treatment. Note the extensive interstitial fibrosis represented by the blue stains. Scale bars, 100 μm. **c** Immunofluorescent staining of apoptotic CMs in cardiac microtissue with an apoptosis-specific marker (Cleaved caspase 3; Cl-Casp3) and cardiac specific marker (sarcomeric-alpha actinin; SAA). White arrow indicates cells co-stained with SAA and Cl-Casp3. **d** Percentage of apoptotic CMs by quantifying ratio of Cl-Casp3 positive cells per number of DAPI-stained cells. Data are the means±SD of replicates (*n* = 3). **p < 0.01. **e** Average diameters of cardiac spheroids. Data are the means±SD of replicates (*n* ≥ 11). **p < 0.01. **f** Immunoperoxidase staining of myofibroblast-specific marker (alpha-smooth muscle actin; α-SMA) in cardiac microtissues after TGF-β1 treatment. **g** CM-MSC cardiac microtissue sections were co-immunostained with anti-vimentin (green) and anti-α-SMA (red). Cells undergoing fibroblast-to-myofibroblast transdifferentiation were positive for both vimentin and α-SMA (arrows). Nuclei were stained with DAPI (blue). Scale bars, 50 μm

To develop a fibrosis model using CM-MSC microtissues, they were treated with the pro-fibrotic growth factor TGF-β1 for 2 weeks. Treatment of TGF-β1 induced irregular beating patterns in cardiac microtissues (Fig. 5a and Additional file 5: Movie 4). To observe collagen deposition, Masson’s Trichrome staining was performed in sections of TGF-β1-treated CM-MSC microtissues in which a thicker collagen layer was detected after increasing the days of TGF-β1 treatment as seen in fibrotic disease tissues (Fig. 5b). A highly consistent pattern of results was obtained by conducting repetitive experiments (Additional file 6: Figure S2). CMs of CM-MSC cardiac microtissues treated with TGF-β1 exhibited significantly increased apoptosis as evidenced by increasing SAA/cleaved caspase-3 double-positive cells (Fig. 5c, d). The average sphere size was found to be approximately 10% smaller in TGF-β1-treated CM-MSC microtissues (554.17 μm ± 14.87) compared with the control (610.36 μm ± 8.06) (Fig. 5e), which is the result of CM apoptosis. Furthermore, TGF-β1 treatment also promoted the transdifferentiation of cardiac fibroblasts into myofibroblasts, a major cause of abnormal collagen secretion, as shown by the increased number of α-SMA-positive cells (Fig. 5f). In the TGF-β1-treated group, α-SMA-positive cells increased over time (Fig. 5f). Most importantly, cells that were positive for both vimentin and α-SMA were observed in the TGF-β1-treated group, strongly indicating that cells of fibroblast origin had transdifferentiated into myofibroblasts (Fig. 5g, arrows). These results demonstrated that treatment of TGF-β1 in CM-MSC cardiac microtissues can recapitulate collagen deposition, enhanced apoptosis in CMs, and myofibroblast differentiation, which are typical pathological phenotypes observed during in vivo myocardial fibrosis.

**Fig. 6:**
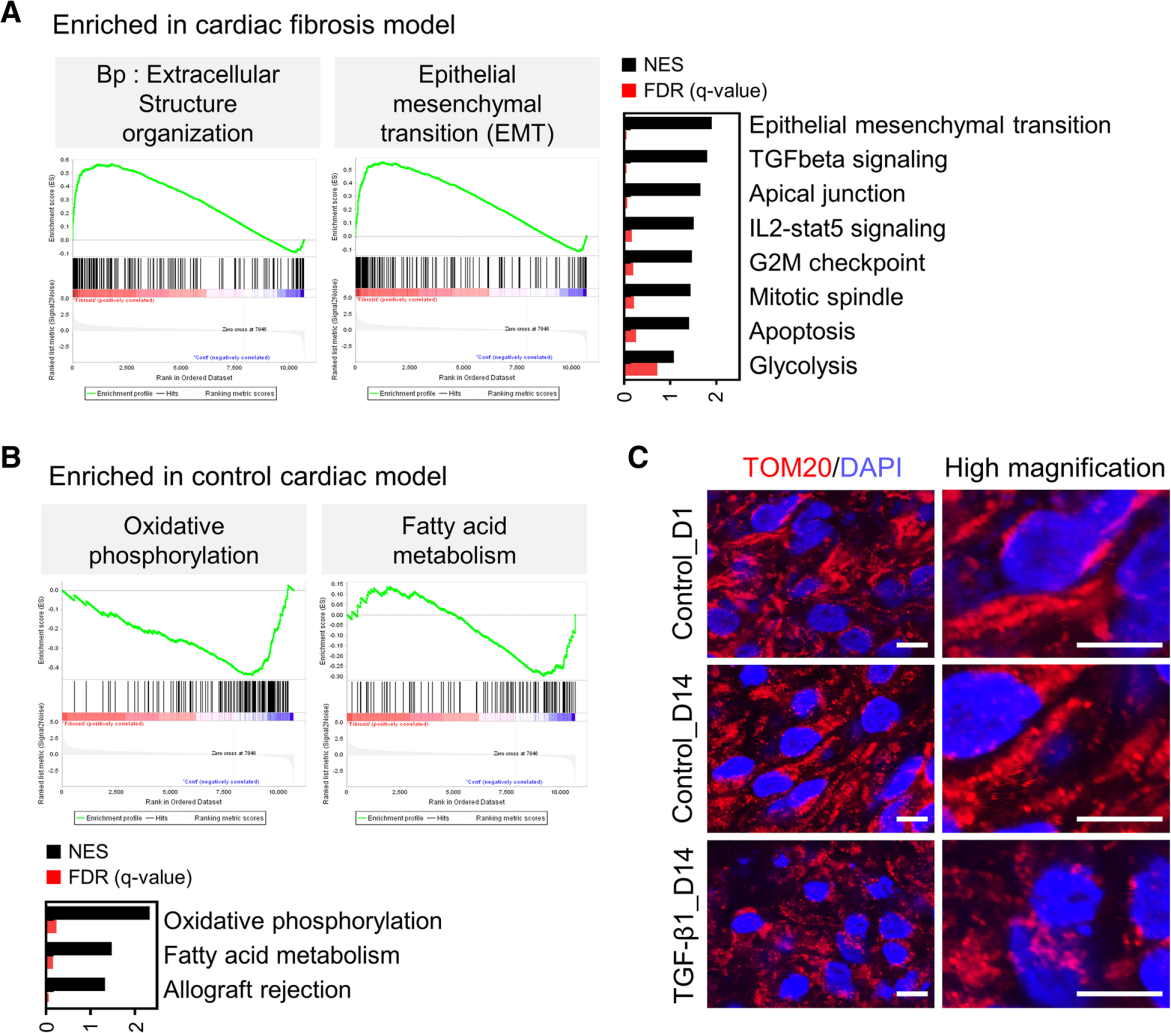
Molecular signatures of TGF-β1-induced cardiac fibrosis models using 3D cardiac microtissue. **a** List of gene set enrichment subsets in TGF-β1 induced cardiac fibrosis model. Enrichment plot of top ranked subset signature by BP (biological process) and hallmarks; extracellular structure organization (BP) and epithelial mesenchymal transition (EMT) (hallmarks) **b** List of gene set enrichment subsets in control cardiac microtissue sample. Enrichment plot of top ranked subset; oxidative phosphorylation and fatty acid metabolism. Normalized enrichment scores (NESs) were calculated to account for differences in gene set size and correlations between gene sets and the expression dataset. The false discovery rate (FDR) q-value represents the probability of false discovery associations for a given NES. FDR q-value lower than 0.25 was considered significant. **c** Immunofluorescence staining of mitochondrial-specific marker (TOM20). Nuclei were stained with DAPI (blue). Scale bars, 10 μm

Furthermore, we analysed global transcription levels to investigate the molecular phenotype of TGF-β1-induced fibrosis in CM-MSC cardiac microtissues. Gene set enrichment analysis (GSEA) by biological process, hallmark and cellular localization was performed to identify functional biological pathways in TGF-β1-induced fibrosis models (Fig. 6 and Additional file 7: Figure S3). Consistent with the cellular phenotype shown in Fig. 5b, the extracellular structure organization was ranked as the upregulation pathway in the biological process analysis (Fig. 6a). Likewise, hallmark analysis showed an increase in gene sets related to epithelial mesenchymal transition (EMT) and TGF-β signalling (Fig. 6a). In agreement with these data, GSEA by the cellular location demonstrated that proteinaceous extracellular matrix or the genes located on the basement membrane were enriched in the TGF-β1-induced fibrosis model (Additional file 7: Figure S3A). These analyses suggested that ECM deformation by TGF-β signalling was a major pathway in the fibrosis model. On the other hand, gene sets related to the respiratory chain or inner mitochondrial membrane proteins were enriched in the control cardiac microtissue model (Additional file 7: Figure S3B). Consistent with these data, GSEA by the hallmark revealed that oxidative phosphorylation and fatty acid (FA) metabolism were decreased in the fibrosis model (Fig. 6b). These results raised the possibility that not only the accumulation of ECM as shown in Fig. 5b but also mitochondrial dysfunction may be induced in the TGF-β1-induced cardiac fibrosis model. To assess this possibility, we performed immunofluorescence of TOM20 to visualize the mitochondrial structure, which is closely related to mitochondrial function. Consistent with previous studies [33, 34], the fragmented mitochondrial morphology was observed in cardiac microtissue treated with TGF-β1, suggesting that mitochondrial dysfunction was induced in the cardiac fibrosis models (Fig. 6c). In addition, as FA metabolism is a predominant metabolic pathway in normal heart [35], changes in the expression of FA metabolism-related genes in fibrosis models may adversely affect the normal function of myocardial cells.

### Drug-induced fibrosis in CM-MSC cardiac microtissues

**Fig. 7:**
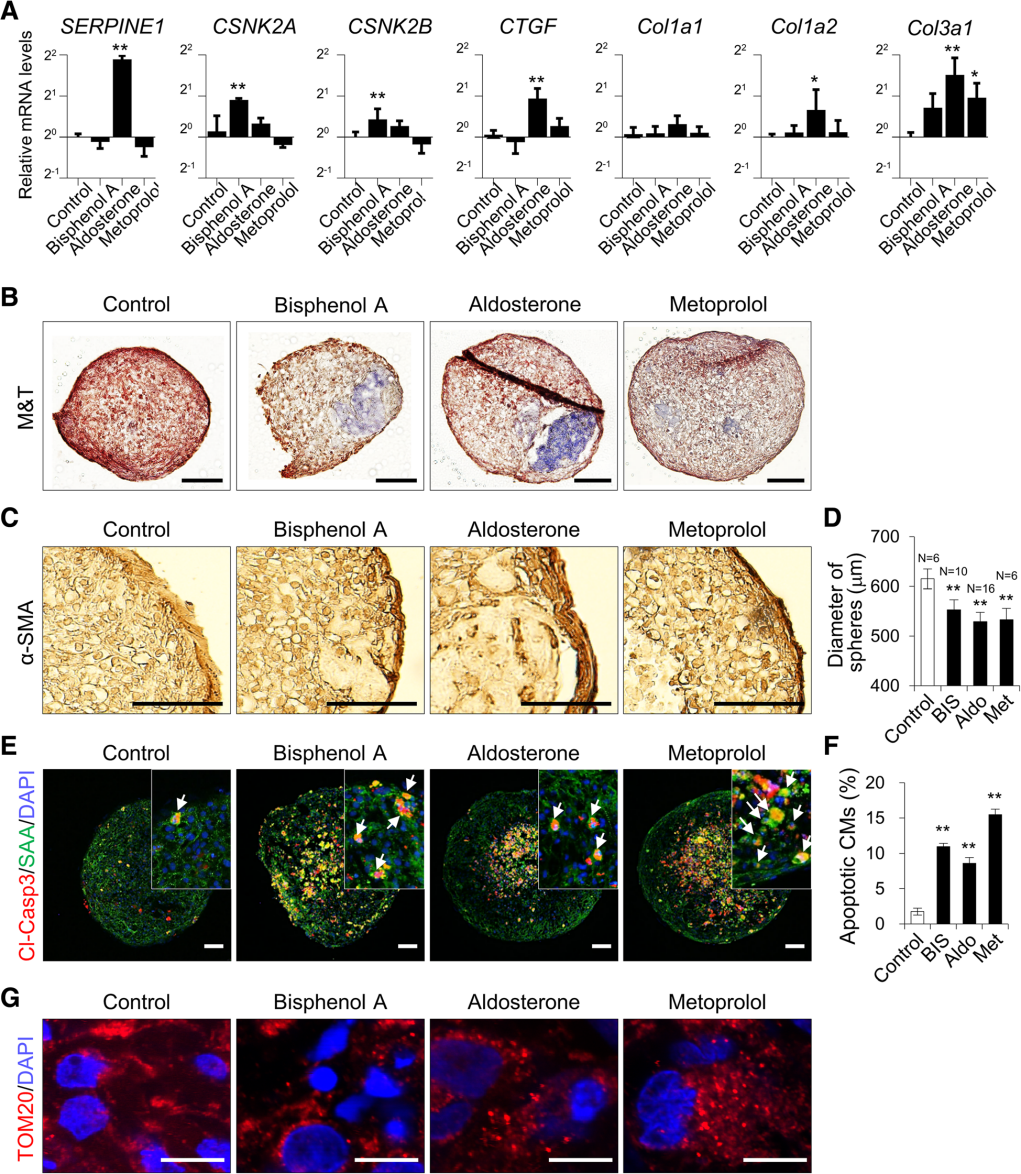
3D cardiac microtissue for in vitro assessment of drug-induced cardiac fibrosis. **a** qRT-PCR analysis for mRNA expression for fibrosis-related collagen genes (*Col1a1*, *Col1a2*, and *Col3a1*) and TGF-β responsive genes (*SERPINE1*, *CSNK2A*, *CSNK2*, and *CTGF*) in CM-MSC cardiac microtissues independently treated with 10 μM concentration of each pro-fibrotic drug for 14 days*.* Data are the means±SD of three independent experimental replicates (*n* = 3). **p < 0.01, *p < 0.05. **b** Masson’s Trichrome staining to detect collagen deposition in CM-MSC cardiac microtissues following treatment with pro-fibrotic drugs for 14 days. Scale bars, 100 μm. **c** Immunoperoxidase staining of myofibroblast-specific marker (alpha-smooth muscle actin; α-SMA) in cardiac microtissues after treatment of pro-fibrotic drugs. Scale bars, 50 μm. **d** Average diameters of cardiac spheroids. Data are the means±SD of replicates (*n* ≥ 6). **p < 0.01. **e** Immunofluorescent staining of apoptotic CMs in cardiac microtissue with an apoptosis-specific marker (Cleaved caspase 3; Cl-Casp3) and cardiac-specific marker (sarcomeric-alpha actinin; SAA). White arrow indicates cells co-stained with SAA and Cl-Casp3. Scale bars, 50 μm. **f** Percentage of apoptotic CMs by quantifying ratio of Cl-Casp3 positive cells per number of DAPI-stained cells. Data are the means±SD of replicates (*n* = 4). **p < 0.01. **g** Immunofluorescence staining of mitochondrial-specific marker (TOM20). Nuclei were stained with DAPI (blue). Scale bars, 10 μm

To further explore the applicability of CM-MSC cardiac microtissues to drug-induced cardiac fibrosis models, we examined reference pro-fibrotic mediators, including bisphenol A, aldosterone and metoprolol, which are known to induce cardiac fibrosis in vivo [36–38]. To explore drug responsiveness in the CM-MSC cardiac microtissue model, tissues were treated with each pro-fibrotic mediator at a concentration of 10 μM for 2 weeks. qRT-PCR showed an increase in TGF-β1-responsive genes, including *SERPINE1*, *CSNK2A*, *CSNK2B*, and *CTGF*, and collagen genes, including *COL1A1*, *COL1A2*, and *COL3A1*, although variations of responsiveness to each drug were observed (Fig. 7a). An increase in collagen deposition was seen in each compound-treated group compared with control CM-MSC cardiac microtissues (Fig. 7b). Hence, the contribution of pro-fibrotic mediators in transdifferentiation into myofibroblasts was examined in CM-MSC cardiac microtissues, as shown by immunohistochemistry with anti-α-SMA antibody (Fig. 7c). Among the three reference compounds, aldosterone was found to be the most effective for inducing fibrosis in our CM-MSC cardiac microtissues. The size of the spheres was significantly reduced after treatment with each compound (Fig. 7d). CMs of CM-MSC cardiac microtissues treated with reference compounds also exhibited significantly increased apoptosis as evidenced by increasing SAA/cleaved caspase-3 double positive cells (Fig. 7e, f). Moreover, mitochondrial phenotypes were confirmed in the drug-treated cardiac microtissues through TOM20 immunohistochemistry, indicating that mitochondrial fragmentation was increased by treatment with each drug (Fig. 7g). We examined whether treatment with pro-fibrotic drugs could directly influence CMs and the mitochondrial structure. There was no apparent apoptotic response or mitochondrial fragmentation in CMs (Additional file 8: Figure S4). Overall, 3D CM-MSC cardiac microtissues were able to recapitulate the pathological phenotypes of cardiac fibrosis, with differences in fibrotic responses depending on the drug. Therefore, the fibrosis model using CM-MSC cardiac microtissues will be an applicable model system for investigating mechanistic insights into fibrotic diseases and in vitro screening of compounds for drug-induced cardiac fibrosis.

## Discussion

The goal of engineering a 3D cardiac tissue model is to provide new opportunities for in vitro cardiac modelling with physiologically relevant cell types and microenvironment. The human 3D cardiac microtissue described in this study is expected to improve our knowledge about clinically relevant information regarding cardiac fibrosis and disclose advanced features of cardiac fibrosis. It is now widely believed that cultures of single cell types are very simplistic models, because they do not mimic the complexity and heterogeneity of human tissues. The normal heart is composed of several different cell types, such as CMs, endothelial cells, smooth muscle cells and fibroblasts, which play a role in the development of the heart and its normal functions [39]. Even within the in vitro environment, non-CMs influence the improvement of CM phenotypes and tissue architecture only in 3D cultures [40, 41]. These results are consistent with observations that most cell types do not exhibit the full spectrum of tissue-specific functionality in 2D cultures, since they do not maintain their phenotypes and patterns of gene expression precisely in the different environments compared with native tissue [42]. Therefore, to mimic the native cardiac tissue, our in vitro 3D co-culture strategy that includes relevant cell types appears most appropriate.

In this study, we have established for the first time a human 3D cardiac microtissue containing hESC-derived CMs in combination with hESC-derived MSCs. During heart development, fibroblasts, which are important cells that produce fibrotic ECM proteins [43], arise from multipotent progenitor cells, especially MSCs [19–21, 23, 44]. The cellular origins of cardiac fibroblasts are varied, including epithelial-mesenchymal transition (EMT) of epicardium cells, endothelial-mesenchymal transition (endo-MT) of epithelial cells, and MSCs [45]. Fibroblasts are connective tissue cells of mesenchymal origin that synthesize and secrete the main components of ECM, such as interstitial collagen and fibronectin [46, 47]. Fibroblasts also play an important role in fibrotic tissue formation by conversion into myofibroblasts under pathological conditions [48]. However, there is currently no standardized protocol for the reliable differentiation of hPSCs into cardiac fibroblasts, whereas, the differentiation methods of hPSCs into MSCs are well established [49, 50]. It has been also reported that MSCs can transdifferentiate into fibroblasts in vitro and in vivo [51]. Cardiac MSCs play an important role in preserving normal cardiac functions, as well as in cardiac remodelling at various pathological stages, by responding early to myocardial infarction [52]. Therefore we developed 3D cardiac microtissues consisting of CMs in combination with MSCs as a source of fibroblasts for developing the in vitro cardiac fibrosis model. The generation of cardiac spheroids with hESC-derived CMs and MSCs has an advantage in terms of the spheroid formation time. As shown in Fig. 3a, aggregates started to form only 1 day after seeding CMs mixed with 20% MSCs, and compact and circular spheroids were successfully generated, whereas loose aggregates with poor cell-cell contacts were formed when CM was cultured only in 3D. Consistent with our findings, Ong et al. have shown that multicellular cardiac spheres are generated from hiPSC-CMs only when they are mixed with human adult ventricular cardiac fibroblasts and human umbilical vein endothelial cells [53]. Furthermore, increased expression of sets of genes associated with ECM organization and cell adhesion (Fig. 4d), which are known to be essential for sphere formation [54], in CM-MSC cardiac spherical microtissues allows more rapid sphere formation in comparison to CM spheroids.

DDR2 is known to be selectively expressed in cardiac fibroblasts [45, 46, 55], although it appears to be also expressed in pericytes/vascular smooth muscle cells [56]. As shown in Fig. 4b, all of vimentin-positive MSCs were not co-expressed with DDR2, whereas some cells within CM-MSC cardiac spheroids were double-positive for vimentin and DDR2, suggesting a transdifferentiation of MSCs into fibroblasts within CM-MSC cardiac spheroids. Our findings are supported by a report that CD44-positive MSCs served as a major precursor pool for fibroblasts that mediate scar formation and wound healing after myocardial infarction [18]. In addition, bone marrow-derived MSCs [57, 58] and perivascular-resident MSCs [59, 60], as well as cardiac fibroblasts, which are known to be direct sources of myofibroblasts and MSCs, also exhibit myofibroblastic phenotypes in vitro in response to TGFβ treatment [61].

As in previous cardiac fibrosis models, mouse and rat neonatal CMs are commonly used for developing engineered cardiac tissue because terminally differentiated adult CMs cannot proliferate sufficiently in vitro [8, 10, 12]. In this study, CMs derived from hESCs were well characterized by the expression of cardiac function-related proteins (Fig. 1a-e). Particularly, CMs were connected to each other by the intercalated disc structure by using immunofluorescence staining of Cx43, the most abundant cardiac gap junction protein (Fig. 1e). Cx43 expression was observed as dots in the intercellular connected regions, in which the ends of myofibrils were connected, consistent with a previous study [62]. hPSC-derived CMs have been widely used to perform drug and toxicity testing in vitro [63, 64]. Furthermore, CMs differentiated from hiPSCs generated from patients with genetic cardiac diseases also allow for the generation of CMs carrying the genetic background of a given patient, creating highly tailored models for cardiac diseases in vitro [65]. Recently, a cardiac model of multicellular systems with several cell types, including CMs, endothelial cells, and fibroblasts, was developed to engineer a physiologically relevant cardiac tissue model using a 3D culture system, various biomaterials and bioprinting technology [10, 13, 53, 66–69]. These multicellular spheroids and 3D models will be applied for developing cardiac fibrosis models by introducing external fibrotic signals. Moreover, when developing in vitro cardiac fibrosis models for personalized drug efficacy and toxicity testing, the iPSC technology applied in this study enables the creation of in vitro disease models in which various cell types with the same genetic background can be assembled. Therefore, disease models using patient-specific cells can be used as platforms to explore new therapies by providing individually optimized treatments.

In this work, we established a 3D in vitro model of cardiac fibrosis using 3D cardiac microtissues by treatment with TGF-β1, which is a potent stimulator that promotes the differentiation and proliferation of myofibroblasts during the course of this disease [70]. Our in vitro model, long-term treatment of TGF-β1 induces irregular beating patterns (Fig. 5a), an increase in the accumulation of fibrillar collagen deposition (Fig. 5b), elevated expression of α-SMA, conversion to myofibroblasts (Fig. 5f), increase in the rate of apoptosis of CMs (Fig. 5c, d), and disruption of the mitochondrial network as shown by transformation of filamentous into punctate mitochondria (Fig. 6c), consistent with the pathological changes during cardiac fibrosis [70, 71]. However, we did not detect increased proliferation of MSC or transdifferentiated myofibroblasts in response to TGF-β1 treatment (data not shown), which may have been due to the inclusion of serum-free medium in the cardiac sphere culture conditions. Moreover, we examined whether 3D cardiac microtissues could be used to detect and monitor cardiac fibrosis using known pro-fibrotic mediators. After a 2-week exposure to pro-fibrotic mediators, including bisphenol A, aldosterone and metoprolol, 3D cardiac microtissues display fibrotic features (Fig. 7a-g), such as an up-regulation of pro-fibrotic and TGF-β1-responsive genes, collagen secretion and deposition; increased α-SMA expression; and disorganization of the mitochondrial network, leading to mitochondrial fragmentation, without significant differences compared to TGF-β1-treated cardiac microtissues. These results suggested that our 3D cardiac model can be applied to identify thus far unknown fibrotic compounds and to provide a platform for studying mechanisms of cardiac fibrosis. However, the expression levels of TGF-β1 responsive genes varied among pro-fibrotic drugs (Fig. 7a), suggesting that there may be different detailed molecular mechanisms for the activation of the TGF-β1 pathway. The link between pro-fibrotic drugs and the TGF- β1 pathway is very complex since different molecular mechanisms are involved. Therefore, this research model could provide the basics for studying the development of cardiac fibrosis in response to the different drugs.

However, we recognize the limitations of this in vitro model in recapitulating human diseases, as it lacks adequate blood flow and other physiological factors, such as endothelial and inflammatory cells, which are found in the native heart. There is also a need to apply natural and synthetic biomaterials mimicking native ECMs, as biomaterials play a major role in constructing 3D tissue models via supporting cell attachment and alignment and providing tissue-relevant stiffness [72]. Therefore, the further introduction of various cell types present in the heart, application of microfluidics, and use of tissue engineering strategies can be exploited in the development of physiologically relevant in vitro human heart tissue.

## Conclusions

In this study, we report a simplified cardiac microtissue comprising CMs and MSCs derived from hESCs to study cardiac fibrosis in a 3D spheroid platform. Our study highlights the importance of increasing the cellular complexity by adding the appropriate amount of CD44-positive MSCs which can contribute to cardiac fibrosis by generating fibroblasts to better model human cardiac tissues in vitro*.* Because our cardiac tissue model can be adapted to mimic various aspects of cardiac fibrosis, it cannot only be used to provide further insights into the mechanisms underlying cardiac fibrosis but can also potentially contribute to the development of in vitro assay systems for testing pro-fibrotic compounds and new anti-fibrotic therapies.

## Methods

### Cell culture

H9 hESCs were obtained from the WiCell Research Institute (Madison, WI, USA) and maintained as described previously [73]. This study using human embryonic stem cell lines was approved by the Public Institutional Bioethics Committee designated by the Ministry of Health and Welfare (MoHW) (Seoul, Republic of Korea; IRB no. P01–201409-ES-01).

### Differentiation of hESCs into human CMs

Human cardiomyocytes were differentiated as described previously [24]. hESCs were transferred to Matrigel (BD Bioscience, San Jose, CA)-coated plates with mTESR medium (Stem Cell Technologies, Vancouver, Canada) without feeder cells; then, cells were grown to 90% confluency. For mesoderm induction, hESCs were treated with 6 μM CHIR99021 (Tocris, Bristol, UK) for 2 days in Cardiac Differentiation Medium (CDM) consisting of RPMI1640 supplemented with 50 μg/ml L-ascorbic acids (Sigma-Aldrich, St. Louis, MO, USA) and 500 μg/ml recombinant albumin (Sigma-Aldrich). Cardiac specification was performed by treating with 2 μM of Wnt-C59 (Selleck Chemicals, Houston, TX, USA) for another 2 days in CDMs. Subsequently, the cells were replaced with CDM medium once every two days until beating CMs were observed. Differentiated CMs were maintained in RPMI-B27 medium consisting of RPMI1640 supplemented with 50 μg/ml L-ascorbic acids and 1XB27 (Thermo Fisher Scientific, Waltham, MA, USA).

### Differentiation of hESC into human MSCs

Human MSCs were differentiated from hESCs as described previously [74]. hESCs were transferred to Matrigel-coated plates with mTESR medium (Stem Cell Technologies) without feeder cells; then, cells were grown to 50% confluency. For mesenchymal differentiation, medium was changed to MEM-alpha supplemented with 10% FBS and 5 ng/ml bFGF (R&D Systems, Minneapolis, MN, USA) once every two days. Cells were transferred to a gelatine-coated dish every week.

### Generation of CM-MSC cardiac microtissues

The differentiated CM and MSC cells were dissociated into single cells by 0.25% trypsin treatment, and the number of cells was counted with a haemocytometer. The CMs and MSCs were mixed at a ratio of 4:1, and 50,000 cells per well were seeded in a 96 well ultra-low attachment plate with RPMI-B27 media supplemented with 10% FBS. After the sphere formation was completed within 2–3 days, the medium was replaced with RPMI-B27 and the medium was replaced every 2–3 days. To develop the in vitro fibrosis model, 5 ng/ml TGF-β1 or 10 μM concentration of each cardiotoxic chemical, including Bisphenol A, Aldosterone, and Metoprolol (all reagents from Sigma-Aldrich) were treated independently with the indicated concentration of drugs as described in previous reports [36–38] and the samples were harvested for analysis after 2 weeks.

### FACS analysis

To verify the CM and MSC differentiation efficiency, FACS analysis was performed as described previously [75]. The differentiated CMs and MSCs were treated with 0.25% trypsin, dissociated into single cells, and incubated in dPBS containing 2 mM EDTA and 2% FBS for 10 min. After that, antibodies to the surface markers of each cell (Additional file 9: Table S1) were diluted 1:50 and reacted for 30 min. After washing twice with FACS buffer, FACS analysis was performed using Accuri C6 flow cytometry (BD Biosciences) and analysed using FlowJo V10 software (TreeStar, USA).

### Processing of cardiac microtissue, immunostaining and MT staining

Cardiac microtissues were fixed by 4% paraformaldehyde (PFA), cryo-protected in 10%~ 30% sucrose solution and frizzed after embedding in optimal-cutting-temperature (OCT) compound (Sakura Finetek, Tokyo, Japan) as described previously [76]. Frozen section were prepared by cutting at a thickness of 10–15 μm using a cryostat microtome. The frozen sections were permeablized with 0.1% Triton X-100 for immune-staining, blocked with 3% BSA, and incubated with the primary antibody (Additional file 9: Table S1). For immunofluorescence detection, cells were incubated with a fluorescence-conjugated secondary antibody. For immunoperoxidase detection, the sections were incubated with the biotinylated secondary antibody and then followed using the VECTASTAIN elite ABC kit (Cat. NO. PK-6100, vector laboratories) and DAB substrate kit (Cat. NO. SK-4100, Vector Laboratories, Burlingame, CA, USA). Staining of collagen fibres was performed using Masson’s Trichrome staining kit (Cat no. 25088–1, Polysciences, Inc. Warrington, USA).

### Quantitative RT-PCR (qPCR)

To compare mRNA expression levels, total RNA was extracted according to the manufacturer’s protocol using the easyBLUE RNA extraction kit (iNtRON Biotechnology, Inc., Republic of Korea). cDNA was synthesized using the SuperScript™ IV First-strand Synthesis System (Cat.NO.1891050, Thermo Fisher Scientific), and the cDNA was used as a template for real-time qPCR. PCR reactions were performed three times independently using Fast SYBR ™ Green Master Mix (4,385,612, Applied Biosystems, Foster City, CA, USA). The sequence of the gene-specific primers are presented in the Additional file 10: Table S2.

### Transcriptome analysis by microarray

For global transcriptome analysis, cDNA microarray was performed using an Agilent Human GE (V2) 4 X 44 K chip as described previously [77]. Data were normalized by using GeneSpring software, and differentially expressed genes (DEGs) were analysed by MultiExperiment Viewer software (MeV version 4.9.0; http://mev.tm4.org/). Pathway enrichment analysis was performed by Reactome FI plugin of the Cytoscape software (Version 3.2.0, www.cytoscape.org), and gene set enrichment analysis was performed by GSEA 2.2.4 (http://software.broadinstitute.org/gsea/index.jsp). The H (hallmark genesets) subsets of MSigDB (50 gene sets), C5_BP (GO biological process, 4436 gene sets) and C5_CC (GO cellular component, 580 gene sets) were used in this study [78].

### Beating rate analysis of CM-MSC microtissue by tracing of microscopic video files

Beating rates of CM-MSC microtissue were recorded on a microscope (IX83, Olympus, Japan) with an on-stage incubator (Live Cell Instrument, Seoul, South Korea) at 37 °C and 5% CO_2_ to maintain the physiological condition. The plot z-axis profile of each region of interest (ROI) was calculated by Image J software (https://imagej.nih.gov/ij/) program [79].

### Field potential recordings using a multi-electrode array (MEA) system

The hESC-derived CMs were plated on a Matrigel-coated 12-well MEA plate (4 × 10^4^ cell per well) (AXION, Atlanta, GA, USA) in RPMI-B27 media supplemented with 5% FBS. At 24 h after seeding, the medium was replaced with RPMI-B27. Field potentials were recorded at day 6 after seeding using Axion BioSystems’ Maestro MEA systems set as 37 °C and perfused with 5% CO^2^, 20% O^2^, and 75% N^2^. Field potential signals were recoded with Axion BioSystems’ Integrated Studio (AxIS) software version 2.3 and analysed with the Axion Cardiac Data Plotting Tool.

### Statistical analysis and graph drawing

Whether treatments produce significantly different results was evaluated by statistical analysis. The unpaired t-test (Figs. 1c, 2c, 3b, e, 4e, 5d, and e) and one-way ANOVA followed by Dunnett’s multiple comparisons test (Fig. 7a, d, and e) were performed using GraphPad Prism version 6.00 for Windows (GraphPad Software, Inc., USA).

## Additional files

Additional file 1: **Movie 1.** Cardiomyocytes derived from hESCs. (MP4 9007 kb)

Additional file 2: **Figure S1.** Effect of TGF-β1 in CM spheroid. (A) Representative morphology of hESC-derived CM spheroids with or without TGF-β1 treatment for 3 weeks. (B) Masson’s Trichrome staining to visualize collagen fibers in CM spheroids. Scale bars, 100 μm. (MP4 4521 kb)

Additional file 3: **Movie 2.** Cardiac spheroid of hESC-derived CMs on day 14. (MP4 4700 kb)

Additional file 4: **Movie 3.** Cardiac spheroid comprising both hESC-derived CMs and MSCs on day 14. (MP4 1623 kb)

Additional file 5: **Movie 4.** TGF-β1-treated cardiac spheroid comprising both hESC-derived CMs and MSCs on day 14. (MP4 1768 kb)

Additional file 6: **Figure S2.** Collagen deposition in TGF-β1 treated CM-MSC microtissue. Masson’s Trichrome staining to visualize collagen fibres in multiple sections of CM spheroids at 14 days after 5 ng/ml TGF-β1 treatment. Scale bars, 100 μm. (TIF 5720 kb)

Additional file 7: **Figure S3.** Comparative cellular component analysis of control and TGF-β1-induced fibrosis models. Gene set enrichment analysis (GSEA) of transcriptome data in TGF-β1 induced fibrosis model was performed by MSigDB of GO cellular component (580 gene set). (A) List of gene sets enriched in cardiac fibrosis model was shown by normalized enrichment score (NES) and false discovery rate (FDR). Enrichment plot of top ranked subset; proteinaceous extracellular matrix and basement membrane. (B) List of gene sets enriched in control was shown by NES and FDR value. Enrichment plot of top ranked subset, respiratory chain and inner mitochondrial membrane protein complex. (TIF 2203 kb)

Additional file 8: **Figure S4.** Treatment of hESC-derived CMs with pro-fibrotic drugs. (A) Immunofluorescent staining of apoptotic CMs with an apoptosis-specific marker (Cleaved caspase 3; Cl-Casp3). Scale bars, 50 μm. Percentage of apoptotic CMs by quantifying ratio of Cl-Casp3 positive cells per number of DAPI-stained cells. C) Immunofluorescence staining of mitochondrial-specific marker (TOM20). Nuclei were stained with DAPI (blue). Scale bars, 10 μm. (TIF 5406 kb)

Additional file 9: **Table S1.** List of the antibodies used in this study. (DOCX 16 kb)

Additional file 10: **Table S2.** List of the primers used in this study. (DOCX 16 kb)

## Abbreviation

2D: Two dimensional
3D: Three dimensional
CDM: Cardiac differentiation medium
CM: Cardiomyocyte
cTnT: Cardiac muscle troponin T
Cx43: Connexin 43
DEG: Differentially expressed gene
ECM: Extracellular matrix
EMT: Epithelial-mesenchymal transition
Endo-MT: Endothelial-mesenchymal transition
FA: Fatty acid
FP: Field potential
FPD: Field potential duration
GSEA: Gene set enrichment analysis
hESC: Human embryonic stem cell
hiPSC: Human induced pluripotent stem cell
hPSC: Human pluripotent stem cell
MSC: Mesenchymal stem cell
OCT: Optimal-cutting-temperature
PC1: Principal component 1
PC2: Principal component 2
PFA: Paraformaldehyde
SAA: Sarcomeric alpha-actinin
TGF-β1: Transforming growth factor β1
α-SMA: α-smooth muscle actin
