# Supplementary figures and images for: Modelling cardiac fibrosis using three-dimensional cardiac microtissues derived from human embryonic stem cells
Source: J Biol Eng. 2019 Feb 13;13:15. doi: 10.1186/s13036-019-0139-6 (PMC6375184; doi:10.1186/s13036-019-0139-6)

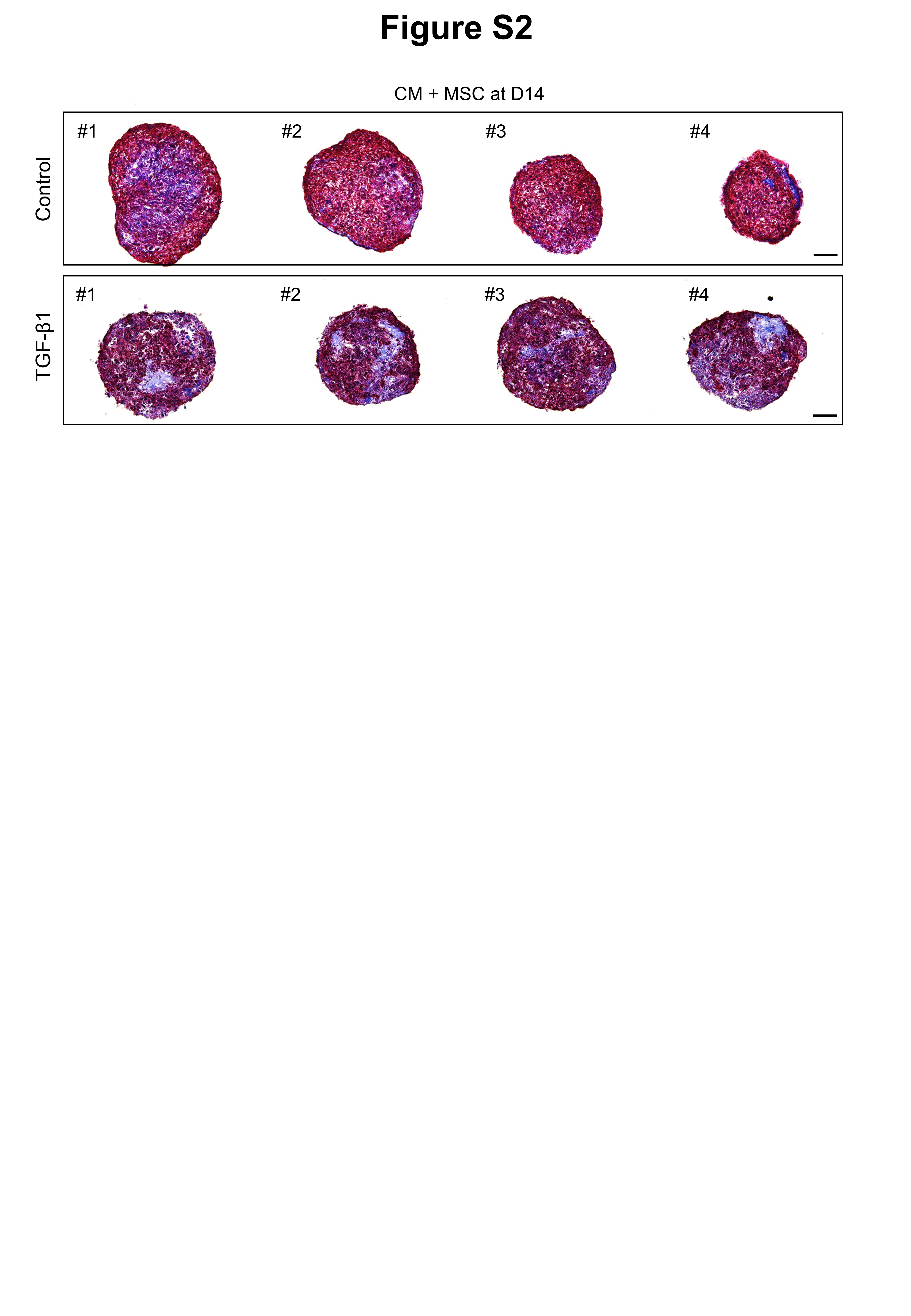

Supplement: Supplementary file 6 — Figure S2. Collagen deposition in TGF-β1 treated CM-MSC microtissue. Masson’s Trichrome staining to visualize collagen fibres in multiple sections of CM spheroids at 14 days after 5 ng/ml TGF-β1 treatment. Scale bars, 100 μm. (TIF 5720 kb) [file 13036_2019_139_MOESM6_ESM.tif]

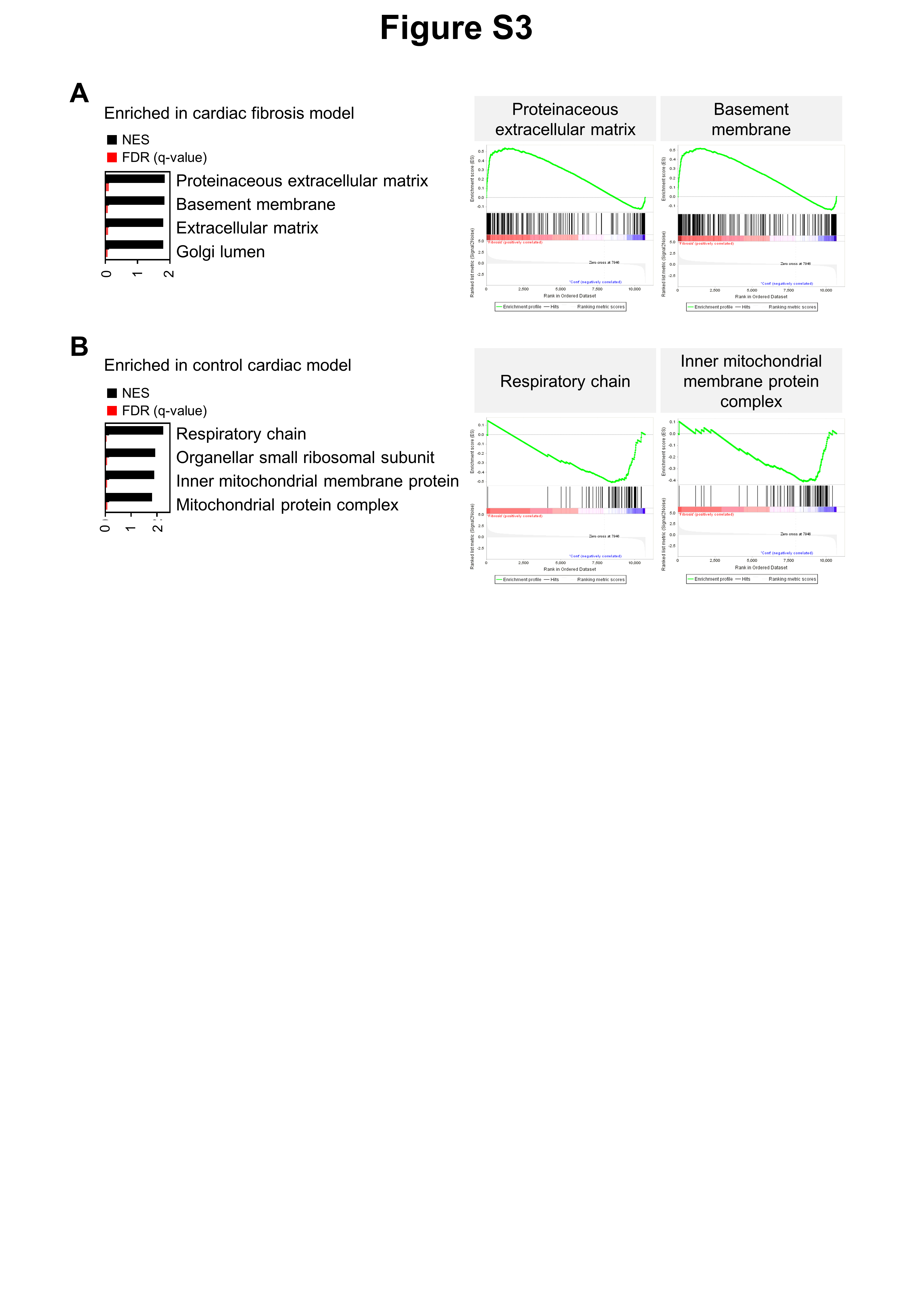

Supplement: Supplementary file 7 — Figure S3. Comparative cellular component analysis of control and TGF-β1-induced fibrosis models. Gene set enrichment analysis (GSEA) of transcriptome data in TGF-β1 induced fibrosis model was performed by MSigDB of GO cellular component (580 gene set). (A) List of gene sets enriched in cardiac fibrosis model was shown by normalized enrichment score (NES) and false discovery rate (FDR). Enrichment plot of top ranked subset; proteinaceous extracellular matrix and basement membrane. (B) List of gene sets enriched in control was shown by NES and FDR value. Enrichment plot of top ranked subset, respiratory chain and inner mitochondrial membrane protein complex. (TIF 2203 kb) [file 13036_2019_139_MOESM7_ESM.tif]

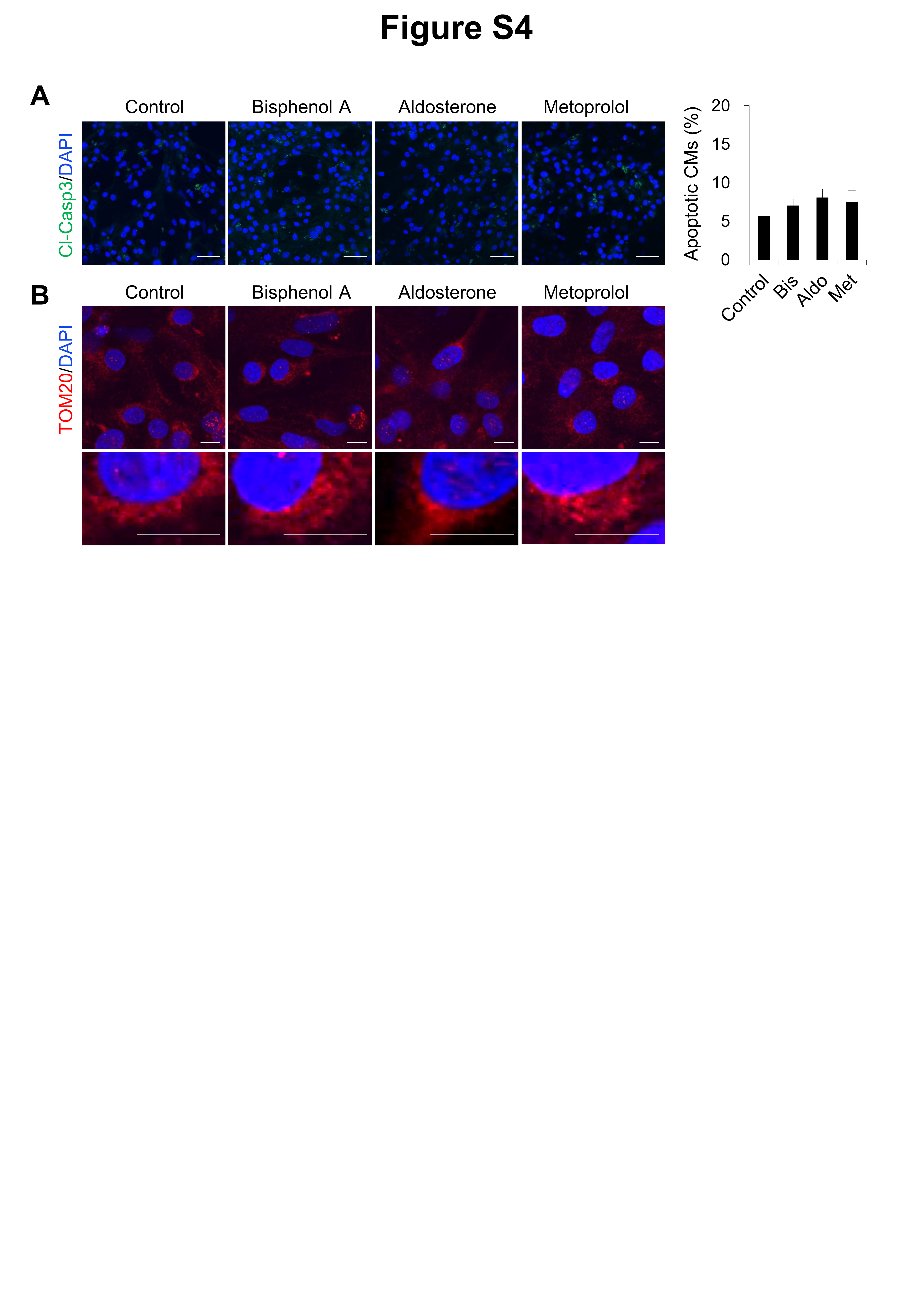

Supplement: Supplementary file 8 — Figure S4. Treatment of hESC-derived CMs with pro-fibrotic drugs. (A) Immunofluorescent staining of apoptotic CMs with an apoptosis-specific marker (Cleaved caspase 3; Cl-Casp3). Scale bars, 50 μm. Percentage of apoptotic CMs by quantifying ratio of Cl-Casp3 positive cells per number of DAPI-stained cells. C) Immunofluorescence staining of mitochondrial-specific marker (TOM20). Nuclei were stained with DAPI (blue). Scale bars, 10 μm. (TIF 5406 kb) [file 13036_2019_139_MOESM8_ESM.tif]
