# Supplementary material for: Modelling cardiac fibrosis using three-dimensional cardiac microtissues derived from human embryonic stem cells
Source: J Biol Eng. 2019 Feb 13;13:15. doi: 10.1186/s13036-019-0139-6 (PMC6375184; doi:10.1186/s13036-019-0139-6)
Supplement: Supplementary file 9 — Table S1. List of the antibodies used in this study. (DOCX 16 kb) [file 13036_2019_139_MOESM9_ESM.docx]

**Supplementary Table 1.** List of antibodies used in this study

|  | Company | Catalog number |
| --- | --- | --- |
| Anti-GATA4 Ab | Santa cruz biotechnology | sc-25310 |
| Anti-MESP1 Ab | Aviva | ARP39374-P050 |
| Anti-NKX2.5 Ab | Abcam | AF2444 |
| Anti-SAA Ab | Abcam | ab9465 |
| Anti-TNNT2 Ab | Abcam | ab64623 |
| Anti-MLC2A Ab | Synapticsystem | 311011 |
| Anti-CX43 Ab | Abcam | ab11370 |
| Anti-MYL2 Ab | Protein tech | 10906-1-ap |
| Anti-CD90 Ab | R&D Systems | MAB2067 |
| Anti-CD105 Ab | R&D Systems | MAB10971 |
| Anti-STRO-1 Ab | R&D Systems | MAB1038 |
| Anti-CD44 Ab | Abcam | ab24054 |
| Anti-Vimentin Ab | CST | 5741s |
| Anti-DDR2 Ab | Abcam | ab63337 |
| Anti-TOM20 Ab | Santa cruz biotechnology | sc-17764 |
| Anti-cleaved caspase 3 Ab | CST | 9664s |
| Anti-alpha SMA Ab | R&D system | MAB1420 |
| PE, anti-Cardiac Troponin T Ab | BD Pharmingen | 564767 |
| PE, anti-CD73 Ab | Miltenyi Biotec | 130-097-943 |
| FITC, anti-CD44v6 Monoclonal Ab | Thermo Scientific | MA5-16966 |
