# Supplementary material for: Modelling cardiac fibrosis using three-dimensional cardiac microtissues derived from human embryonic stem cells
Source: J Biol Eng. 2019 Feb 13;13:15. doi: 10.1186/s13036-019-0139-6 (PMC6375184; doi:10.1186/s13036-019-0139-6)
Supplement: Supplementary file 10 — Table S2. List of the primers used in this study. (DOCX 16 kb) [file 13036_2019_139_MOESM10_ESM.docx]

**Supplementary Table 2.** List of the primers used in this study.

| Gene | Primer (Forward) | Primer (Reverse) |
| --- | --- | --- |
| *GAPDH* | TGTCCCCACTGCCAACGTGTCA | AGCGTCAAAGGTGGAGGAGTGGGT |
| *POU5F1 (OCT4)* | GAGAAGGATGTGGTCCGAGTGTG | CAGAGGAAAGGACACTGGTCCC |
| *TNNT2* | AGGGAGAGCAGAGACCATGT | TTTGGACTCCTCCATTGGGC |
| *TBX5* | CCTCCTCTCCCCCTTCCTAA | TCCGTCTTCGCCTATCAGTG |
| *MYH6* | ACCTGTCCAAGTTCCGCAAG | AGGTTGGCAAGAGTGAGGTT |
| *MYH7* | GGAGTTCAAAGAAGCCTTCAGC | AAAGAGCGTGAGGAAGACGG |
| *ANF* | CCTAAAAAGCAAGCTGAGGGC | ACAGGAGCCTCTTGCAGTCT |
| *ENG (CD105)* | CACTAGCCAGGTCTCGAAGG | CTGAGGACCAGAAGCACCTC |
| *NT5E (CD73)* | CGCAACAATGGCACAATTAC | CAGGTTTTCGGGAAAGATCA |
| *CD44* | AAGGTGGAGCAAACACAACC | AGCTTTTTCTTCTGCCCACA |
| *SGCD* | TGTTGCAGTAGGAAGCCAGA | TAGGCATGTGGGGTGGATTT |
| *MYL1* | TGGCACCAAAGAAAGACGTG | AGAGCTCGAAGGACATCACC |
| *SCN7A* | AGTTTTGGCTGGGCCTTATT | ATAGGCCATGGCAAGTATGC |
| *SCN1B* | TGCGCTATGAGAATGAGGTG | ATCTTCTTGACGACGCTGGT |
| *KCNJ2* | ACCGCTACAGCATCGTCTCT | TCCACACACGTGGTGAAGAT |
| *KCNE4* | GCTCCAAGTTCTGTGCTTCC | GTTCAGACTAACCGGCCAAA |
| *SERPINE1 (PAI1)* | CAATCGCAAGGCACCTCTGA | AAACACCCTCACCCCGAAGT |
| *CSNK2A* | TTTCCTGGACAAACTGCTGC | ACATCATATTGGCGCTGCTG |
| *CSNK2B* | TCAAGACACCATCACACGGA | CAGCGAATCGTCTTGACTGG |
| *CTGF* | GGAAAAGATTCCCACCCAAT | TGCTCCTAAAGCCACACCTT |
| *Col1a1* | CAC CCT CAA GAG CCT GAG TC | GTT CGG GCT GAT GTA CCA GT |
| *Col1a2* | GGTGGTGGTTATGACTTTGG | TCTGGGTGGCTGAGTCTCAA |
| *Col3a1* | GCTCTGCTTCATCCCACTATTA | AACATTCTCCAAATG GAATT |
